# Case Report: Overlapping multiple sclerosis and neuropsychiatric systemic lupus erythematosus with positive MOG-IgG: a case initially misdiagnosed as depression

**DOI:** 10.3389/fimmu.2026.1879355

**Published:** 2026-07-20

**Authors:** Wan Wei, Tao Jin, Liuhai Zhang, Yumeng Sun, Yangyang Xu, Xiaoqin Hong

**Affiliations:** 1 Department of Neurology, Hangzhou First People's Hospital (Hangzhou Geriatric Hospital), Hangzhou, China; 2 Department of Respiratory Medicine, Hangzhou First People's Hospital (Hangzhou Geriatric Hospital), Hangzhou, China

**Keywords:** autoimmunity, depression, MOG-IgG, multiple sclerosis, neuropsychiatric systemic lupus erythematosus

## Abstract

We present a 69-year-old female patient who initially manifested with depression and anhedonia, initially misdiagnosed as primary psychiatric illness. She subsequently developed progressive gait instability and cognitive decline. After comprehensive clinical and laboratory evaluation, she was finally diagnosed with multiple sclerosis (MS) complicated by neuropsychiatric systemic lupus erythematosus (NPSLE). Brain magnetic resonance imaging (MRI) revealed multifocal white matter lesions consistent with demyelination. Serologic testing demonstrated positivity for antinuclear antibody (ANA), anti-double-stranded DNA (dsDNA), anti-SS-A/Ro, anti-histone, anti-nucleosome, and anti-centromere antibodies. Cerebrospinal fluid (CSF) examination confirmed intrathecal synthesis of immunoglobulin G (IgG), as evidenced by CSF-restricted oligoclonal bands (OCBs). Serum myelin oligodendrocyte glycoprotein immunoglobulin G (MOG-IgG) was positive at a titer of 1:32, whereas aquaporin-4 (AQP4) antibodies were negative. Based on the clinical manifestations, laboratory results and disease progression, the final diagnosis was established as coexisting MS and NPSLE. The patient achieved clinical improvement after treatment with glucocorticoids and hydroxychloroquine. This case highlights the diagnostic challenges posed by overlapping autoimmune central nervous system (CNS) disorders and underscores the importance of longitudinal assessment in differentiating MS from MOG-IgG-associated disorder (MOGAD) and NPSLE.

## Case presentation

A 69-year-old right-handed woman with no significant past medical history was admitted to a psychiatric hospital in 2020 for persistent depressive symptoms and anhedonia lasting several years. She denied fever, headache, arthralgia, rash, photosensitivity, or neurologic deficits at onset. She was initially diagnosed with major depressive disorder and received various oral antidepressants for six years, but no obvious clinical improvement was observed. During the six years of treatment for depressive disorder in the psychiatric department, no autoimmune serological tests, CSF examination or brain MRI were performed. At that stage, the patient only presented with isolated psychiatric symptoms without systemic or neurological manifestations of systemic lupus erythematosus (SLE). In 2024, the patient developed new-onset gait imbalance and progressive memory impairment, which gradually deteriorated over the next 18 months. By December 2025, she required assistance walking and exhibited clear cognitive deterioration, prompting referral to our tertiary neurology center.

Neurological examination showed the patient was alert and oriented with fluent speech. Pupils were equal and reactive to light, ocular motility was intact without nystagmus or diplopia. Facial sensation and motor function were normal. Limb strength was preserved (Medical Research Council grade 5/5 in all extremities), muscle tone was normal, and sensory examination including proprioception and vibration sense was unremarkable. Deep tendon reflexes were symmetrically brisk, and bilateral Babinski and Chaddock signs were positive. No cerebellar signs were observed on finger-to-nose or heel-to-shin testing. Meningeal signs were absent. There was no skin rash, malar erythema, or joint swelling.

Laboratory tests revealed an elevated erythrocyte sedimentation rate (ESR) of 34 mm/h and C-reactive protein (CRP) of 11.2 mg/L. Immunological tests demonstrated high-titer ANA (1:1000), positive anti-dsDNA IgG (199.18 IU/mL), as well as positive anti-SS-A/Ro, anti-histone, anti-nucleosome and anti-centromere antibodies. Serum complement C3 decreased to 0.53 g/L, while C4 and C5 remained within normal ranges. Antiphospholipid antibodies, lupus anticoagulant, anti-neutrophil cytoplasmic antibodies (ANCA) and cryoglobulins were all negative. Thyroid function, tumor markers and screening tests for infectious diseases (including HIV and syphilis) were unremarkable.

CSF examination showed an opening pressure of 160 mmH2O. Total protein and glucose levels were normal, and white blood cell count was 0 cells/μL. The CSF IgG index was elevated to 81.7 mg/L, and OCBs were exclusively detected in the CSF, indicating intrathecal IgG synthesis. Serum MOG-IgG was positive at a titer of 1:32 using a live cell-based assay. The validated positive cut-off value of this assay in our laboratory was a titer≥1:10. Both serum and CSF AQP4 antibodies were negative. Paraneoplastic and neuronal surface antibody panels were also negative. All main laboratory findings are summarized in **Table 1**.

**Table 1:** Summary of main laboratory test results of the patient.

Items	Test results	Reference range
Inflammatory markers		
ESR	34 mm/h	0–20 mm/h
CRP	11.2 mg/L	0–8 mg/L
Autoimmune antibodies		
ANA	1:1000 (speckled pattern)	Negative
Anti-dsDNA IgG	199.18 IU/mL	< 100 IU/mL
Anti-SS-A/Ro, anti-histone, anti-nucleosome, anti-centromere	Positive	Negative
Antiphospholipid antibody, lupus anticoagulant, ANCA	Negative	Negative
Complement		
C3	0.53 g/L	0.70–1.40 g/L
C4, C5	Normal	Normal
Cerebrospinal fluid (CSF)		
Opening pressure	160 mmH_2_O	80–180 mmH_2_O
CSF total protein & glucose	Normal	Normal
CSF white blood cell	0 cells/μL	0–5 cells/μL
CSF IgG index	81.7 mg/L	4.8–58.6 mg/L
Oligoclonal bands	Positive (CSF-only)	Negative
Specific autoantibodies		
Serum MOG-IgG	1:32 (live cell-based assay)	< 1:10 (negative)
AQP4-IgG (serum & CSF)	Negative	Negative

Brain MRI (T2-weighted and FLAIR sequences) demonstrated widespread hyperintensities involving the periventricular and subcortical white matter of bilateral frontal, parietal, temporal, and occipital lobes, centrum semiovale, basal ganglia, and left cerebellar hemisphere, lesions were non-enhancing after gadolinium administration (**Figure 1**). Cervical and thoracic spinal cord MRI revealed no focal demyelinating lesions. Cervical computed tomographic angiography revealed no large-vessel stenosis. Visual and brainstem auditory evoked potentials were normal. Electroencephalography showed no epileptiform activity. Chest CT, echocardiogram, and abdominal ultrasound were unremarkable. The patient’s Mini-Mental State Examination (MMSE) score was 21/30 (adjusted for senior high school education), indicating mild cognitive impairment, particularly in attention and delayed recall.

**Figure 1:**
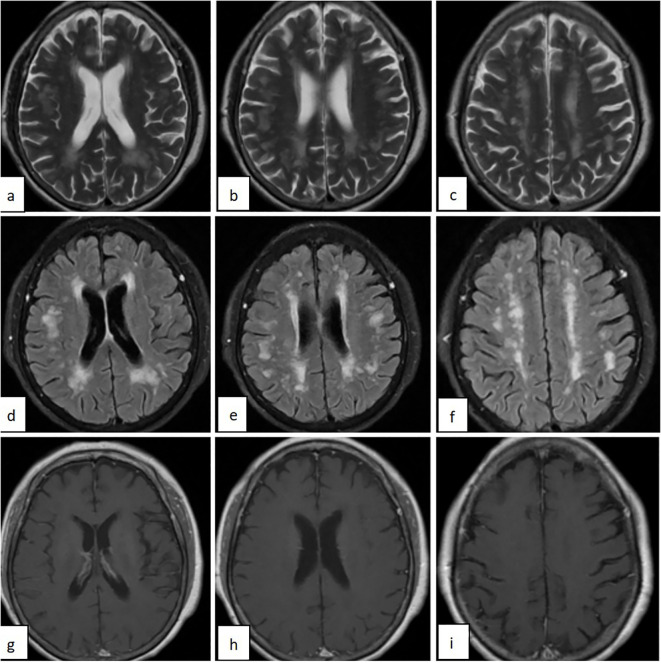
Brain MRI of the patient showed T2-weighted **(a–c)** and FLAIR sequences **(d–e)** widespread hyperintensities involving the periventricular and subcortical white matter of bilateral frontal, parietal, temporal, and occipital lobes, centrum semiovale, basal ganglia, and left cerebellar hemisphere, T1-weighted enhanced showed no lesion enhancement **(f–i)**.

Although no demyelinating lesions were detected on cervical and thoracic spinal cord MRI, brain lesions were widely distributed in the periventricular white matter, juxtacortical areas, infratentorial region (left cerebellar hemisphere) and deep white matter. Lesions involving at least two of the four classic anatomical regions for MS met the criteria for dissemination in space. Given the clinical-radiological evidence of dissemination in space, and CSF-specific OCBs were applied to meet the criterion of dissemination in time (DIT). No gadolinium-enhancing lesions or interval new T2 lesions were detected during follow-up, so progressive neurological symptoms alone were not used as evidence of DIT. Based on above findings, the patient fulfilled the 2017 McDonald criteria (1) for MS. Concurrently, the presence of high-titer ANA, anti-dsDNA, hypocomplementemia, and neuropsychiatric symptoms in the absence of alternative causes met the 2019 EULAR/ACR classification criteria (2)for NPSLE. The patient was diagnosed with coexisting MS and NPSLE, with MOG-IgG likely representing a false-positive or epiphenomenon. Oral prednisone (60 mg/day, tapered over 6 months) and hydroxychloroquine (200 mg twice daily) were initiated. Antidepressants were continued under psychiatric care. At three-month follow-up, the patient reported improvements in mood, mental clarity and gait stability, and her MMSE score increased to 24/30. Follow-up brain MRI showed stable lesions without new or gadolinium-enhancing lesions(**Figure 2**). A timeline figure was added to clearly demonstrate the entire disease course (**Table 2**).

**Figure 2:**
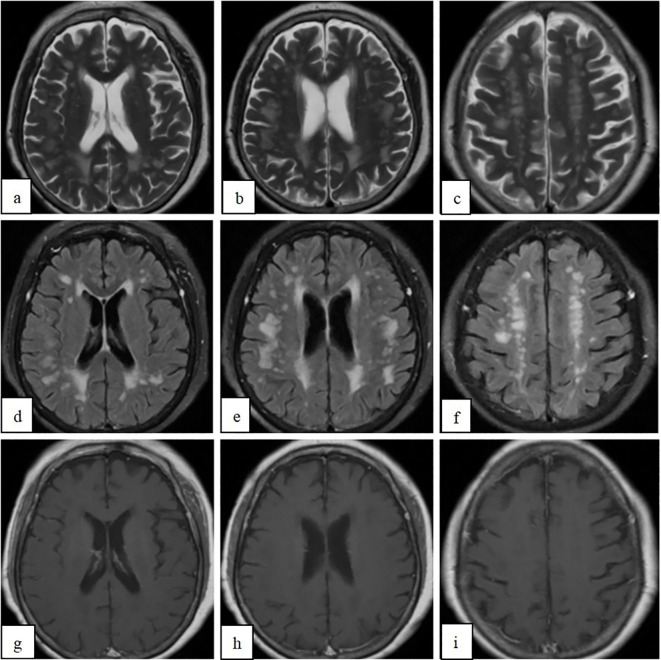
Brain MRI of the patient at 3-month follow-up showed T2-weighted **(a–c)**, FLAIR sequences **(d–e)** and T1-weighted enhanced **(f–i)** stable lesion burden without new or enhancing lesions.

**Table 2:** Timeline of the patient’s clinical course, examinations, treatment and follow-up.

Timeline	Key events
2020	Persistent depression and anhedonia developed. The patient was diagnosed with major depressive disorder in the psychiatric department, and antidepressant treatment was commenced.
2020–2024	Antidepressant treatment with unsatisfactory efficacy; no additional immunological, CSF or neuroimaging tests performed.
2024	Development of progressive gait dysfunction and memory decline.
Dec 2025	Profound cognitive impairment and impaired ambulation; referral to the neurology department.
After admission	Comprehensive laboratory, CSF, neuroimaging and neurophysiological examinations completed.
Diagnosis	Confirmed concurrent MS and NPSLE; serum MOG-IgG titer 1:32 (live cell-based assay).
Treatment initiation	Started on prednisone and hydroxychloroquine; antidepressants continued.
3-month follow-up	Favorable clinical response; brain MRI revealed stable lesions without new or enhancing lesions.

## Discussion

This case presents a complex diagnostic challenge caused by overlapping features of multiple autoimmune CNS disorders, including MS, NPSLE, and MOGAD. The patient initially presented with isolated psychiatric symptoms mimicking primary depression—a recognized prodrome in both MS and NPSLE, leading to a prolonged delay in correct diagnosis. Epidemiological data indicate that neuroinflammatory autoimmune diseases are important differential diagnoses for patients with refractory depression (3). Multiple case series and clinical studies published in psychiatric journals have documented missed diagnoses of NPSLE and MS in patients with isolated depressive symptoms (4–6). For patients, especially female individuals, with newly onset psychiatric disorders refractory to antidepressants or antipsychotics, underlying autoimmune diseases should be fully considered in the differential diagnosis.

Systemic lupus erythematosus(SLE) is a chronic idiopathic autoimmune disease with diverse clinical manifestations involving the skin and mucous membranes, musculoskeletal system, blood system, kidneys and cardiovascular system (7), following a relapsing-remitting course (8). Neuropsychiatric manifestations occur in up to 75% of patients with SLE and define NPSLE when attributable to autoimmune CNS injury (9). These may include mood disorders, psychosis, cognitive dysfunction, seizures, and cerebrovascular events (10).Importantly, NPSLE can precede systemic manifestations by years, complicating early recognition (11). Our patient lacked classic cutaneous, renal, or musculoskeletal features of SLE, yet fulfilled immunologic and clinical criteria for SLE with CNS involvement. The presence of low C3, high-titer ANA and dsDNA, and absence of alternative explanations support primary NPSLE rather than secondary causes including infection, metabolic derangement, drug-induced encephalopathy.

The detection of MOG-IgG at low titer (1:32) adds another layer of complexity. MOGAD typically presents with optic neuritis, transverse myelitis, or acute disseminated encephalomyelitis (ADEM)-like phenotypes, though some patients exhibit MS-like presentations (12). Recent studies emphasize that MOG-IgG seropositivity does not automatically equate to MOGAD, especially when titers are low or clinical/MRI features diverge from typical MOGAD patterns (13). Notably, lesion evolution differs significantly between MS and MOGAD: brain lesions resolve completely in 50–83% of MOGAD cases versus only 1–17% in MS; spinal cord lesions resolve in 79% of MOGAD but rarely in MS (12). Accumulated evidence has confirmed that MOG-IgG positivity can occur as a cross-reactive autoantibody or a secondary epiphenomenon in patients with systemic autoimmune diseases such as SLE (14). Although comorbidity between MOGAD and rheumatic diseases is rare, one previous study reported a MOG-IgG seropositivity rate of 8% among SLE patients (15). In our patient, lack of lesion regression on follow-up MRI argues against MOGAD as the dominant pathology. MOG-IgG may represent an epiphenomenon or cross-reactive autoantibody in the context of broader immune dysregulation.

MS is an autoimmune-related inflammatory demyelinating disorder of the central nervous system (16). Progressive MS includes primary progressive MS (PPMS) and secondary progressive MS (SPMS) (17).different from relapsing-remitting MS (RRMS), progressive MS manifests as persistent neurological deterioration, accumulating disability. A diagnosis of PPMS requires at least one year of continuous neurological progression, plus MRI evidence of demyelination in two or more sites of the brain or spinal cord, or positive CSF-restricted OCBs (1).Typical imaging features of PPMS include prominent brain and spinal cord atrophy, fewer brain lesions but more spinal cord lesions. This subtype causes gradual, irreversible neurological deficits from onset, with no relapses or remissions (17).Approximately 85% of initial MS diagnoses are RRMS, which presents with acute attacks followed by remission and stable phases (18). Although the patient experienced progressive neurological decline for more than one year, her symptoms improved gradually following treatment. No spinal cord involvement was detected, and a distinct remission phase was noted. Consequently, based on the overall clinical profile, we diagnosed the patient with RRMS rather than PPMS. Extended clinical follow-up will be performed to monitor her condition.

Furthermore, distinguishing NPSLE from MS in clinical practice is highly challenging due to overlapping clinical manifestations (19).CNS demyelinating syndrome occurring in the context of SLE may be a manifestation of NPSLE or a comorbidity of NPSLE and MS. Differential diagnosis of these two conditions is clinically important due to differences in treatment regimens. The main differential points are as follows: patients with overlap syndrome tend to have a milder SLE phenotype without major extra-CNS organ involvement, and MS usually follows a relapsing-remitting course with cumulative disability (20). In addition, we further explicitly compare the core distinguishing features among MS, isolated NPSLE and their overlap syndrome from four aspects including MRI manifestations, CSF profiles, serological markers and treatment strategies. First, MRI characteristics (21). Typical MS lesions are predominantly distributed in periventricular white matter, juxtacortical areas and infratentorial regions; spinal cord lesions are common, and chronic lesions rarely resolve completely. For isolated NPSLE, brain lesions are often confined to a single region, mainly involving deep white matter and gray matter with ill-defined boundaries, while spinal demyelinating lesions are uncommon. In overlap syndrome, lesions tend to present the combined features of both diseases, with widespread multi-region involvement as seen in this patient. Second, CSF findings. Although intrathecal IgG synthesis and CSF-restricted OCBs are characteristic CSF features of MS, they lack disease specificity and may be identified in nearly 42–43% of patients with NPSLE (19, 22). Among patients with NPSLE, the elevation of CSF IgG index has a dual origin: endogenous intrathecal IgG synthesis and extravasation of peripheral IgG due to blood-brain barrier (BBB) disruption. Notably, NPSLE demonstrates a far lower frequency of pathological intrathecal IgG synthesis compared with MS. Although MS patients also present with an increased CSF IgG index, this abnormality originates purely from CNS-localized intrathecal IgG synthesis, and BBB integrity is preserved in most affected individuals (22). As elevated CSF IgG index is a shared laboratory feature of the two autoimmune CNS disorders, patients with overlapping NPSLE and MS frequently exhibit a superimposed increase in IgG index. In such complicated cases, differential diagnosis relies on the combined evaluation of serum autoantibody spectra and characteristic MRI manifestations. Third, serological markers. Patients with NPSLE consistently present with positive ANA, anti-dsDNA and hypocomplementemia, which are absent in typical MS. Fourth, treatment approaches. Conventional MS requires long-term disease-modifying therapies (DMTs). Isolated NPSLE is mainly treated with glucocorticoids and antimalarial agents such as hydroxychloroquine. For overlap syndrome, systemic immunosuppression is prioritized to control overall immune activation, and DMTs can be added dynamically according to MS disease activity.

In this case, the patient had a milder SLE phenotype without typical extra-CNS manifestations such as rash, arthralgia and fever. Brain MRI showed lesions involving multiple intracranial regions rather than a single region, with blurred boundaries and variable morphology, and CSF-restricted OCBs were positive, all of which strongly supported the diagnosis of NPSLE-MS overlap. Given the coexistence of two autoimmune disorders requiring systemic immunomodulation, we prioritized systemic immunosuppressive therapy (glucocorticoids combined with hydroxychloroquine) to control NPSLE activity and generalized immune dysregulation. The patient is an elderly female with progressive cognitive impairment and poor general condition. Considering the advanced age, potential adverse effects and drug tolerance of conventional MS disease-modifying therapies (DMTs), we did not initiate DMTs for MS at the current stage. During follow-up, the patient achieved clinical improvement and stable imaging lesions under the current regimen. We will dynamically evaluate her condition and consider adding DMTs if MS-related symptoms deteriorate in the future. We explicitly state that short follow-up duration is a limitation: long-term clinical outcomes, dynamic changes of autoantibodies and long-term imaging prognosis cannot be fully evaluated within 3 months. Longer regular follow-up will be continued for this patient in the future.

## Conclusion

This case demonstrates that overlapping CNS autoimmune disorders, particularly MS and NPSLE, may initially present with isolated psychiatric symptoms that mimic primary mood disorders. Clinicians should maintain a high index of suspicion for underlying neuroinflammatory conditions in patients with refractory depression, especially when accompanied by systemic autoantibodies or subtle neurological signs. Longitudinal integration of clinical, imaging, and laboratory data is crucial for accurate diagnosis. MOG-IgG positivity must be interpreted in clinical context, as it may not always indicate MOGAD. Early immunomodulatory intervention may improve outcomes in these treatable conditions.

## Data Availability

The original contributions presented in the study are included in the article/supplementary material. Further inquiries can be directed to the corresponding author.
